# Efficient Enrichment of Hepatic Cancer Stem-Like Cells from a Primary Rat HCC Model via a Density Gradient Centrifugation-Centered Method

**DOI:** 10.1371/journal.pone.0035720

**Published:** 2012-04-25

**Authors:** Wei-hui Liu, Xing Wang, Nan You, Kai-shan Tao, Tao Wang, Li-jun Tang, Ke-feng Dou

**Affiliations:** 1 PLA Center of General Surgery, General Hospital of Chengdu Army Region, Chengdu, Sichuan Province, China; 2 Department of Hepatobiliary Surgery, Xijing Hospital, Fourth Military Medical University, Xi'an, Shaanxi Province, China; The University of Hong Kong, Hong Kong

## Abstract

**Background:**

Because few definitive markers are available for hepatic cancer stem cells (HCSCs), based on physical rather than immunochemical properties, we applied a novel method to enrich HCSCs.

**Methodology:**

After hepatic tumor cells (HTCs) were first isolated from diethylinitrosamine-induced F344 rat HCC model using percoll discontinuous gradient centrifugation (PDGC) and purified via differential trypsinization and differential attachment (DTDA), they were separated into four fractions using percoll continuous gradient centrifugation (PCGC) and sequentially designated as fractions I–IV (FI–IV). Morphological characteristics, mRNA and protein levels of stem cell markers, proliferative abilities, induced differentiation, *in vitro* migratory capacities, *in vitro* chemo-resistant capacities, and *in vivo* malignant capacities were determined for the cells of each fraction.

**Findings:**

As the density of cells increased, 22.18%, 11.62%, 4.73% and 61.47% of primary cultured HTCs were segregated in FI–FIV, respectively. The cells from FIII (density between 1.041 and 1.062 g/ml) displayed a higher nuclear-cytoplasmic ratio and fewer organelles and expressed higher levels of stem cell markers (AFP, EpCAM and CD133) than cells from other fractions (*P<*0.01). Additionally, *in vitro*, the cells from FIII showed a greater capacity to self-renew, differentiate into mature HTCs, transit across membranes, close scratches, and carry resistance to chemotherapy than did cells from any other fraction; *in vivo*, injection of only 1×10^4^ cells from FIII could generate tumors not only in subcutaneous tissue but also in the livers of nude mice.

**Conclusions:**

Through our novel method, HCSC-like cells were successfully enriched in FIII. This study will greatly contribute to two important areas of biological interest: CSC isolation and HCC therapy.

## Introduction

Over the last decade, the notion that tumors are maintained by their own stem cells, the so-called cancer stem cells (CSCs), has created great excitement in the research community [1]. CSCs are generally dormant or slowly cycling tumor cells that have the ability to reconstitute tumors [1]. They are thought to be involved in tumor resistance to chemo/radiation therapy and tumor relapse and progression. CSCs are important because they are responsible for resistance to treatment. Obtaining further understanding of CSC-specific traits will offer insights regarding the early stages of tumorigenesis to aid in preventing this phenomenon and to enhance the selectivity of antitumor therapies. The existence of CSCs was first proposed over 40 years ago [2]; however, they were only recently isolated from solid tumors, including tumors of the breast [3], prostate [4], brain [5] and colon [6]. Although CSCs have been isolated from some types of tumors, there is much debate surrounding this topic.

Hepatocellular carcinoma (HCC) is a highly malignant type of solid tumor associated with frequent metastasis and poor prognosis [7]. Isolated hepatic CSCs (HCSCs) represent a suitable *in vitro* model for developing therapeutic strategies aimed at eradicating the tumorigenic subpopulation within HCC. It is for this reason that the identification and understanding of HCSCs are crucial. To isolate HCSCs, a variety of separation techniques are available. One common method for isolating CSCs has been to characterize their cell-surface phenotype and use markers to negatively or positively select for particular cells. It is reported hepatic tumor cells (HTCs) with the CD133+ or EpCAM+ phenotype have stem-like properties [8]; however, they have been shown to exhibit limited plasticity. At present, a specific marker for the isolation of HCSCs remains controversial. An alternative method for isolating HCSCs is urgently needed. Another method for CSC separation is based on the differential efflux of fluorescent dyes, such as rhodamine 123 or Hoechst 33342. Recently, the isolation of side population (SP) cells using Hoechst 33342 dye has become a useful method for obtaining CSCs from various tumors. In a previous study by our group, we enriched HCSCs from the MHCC97 cell line based on the efflux of rhodamine 123 or Hoechst 33342 [9]. However, there are still some limitations associated with this method, such as detecting false positive stem cells and the requirement for special instruments. The final option for the isolation of CSCs is based on physical separation methods, such as density gradient separation. In a previous study, we successfully isolated fetal liver stem/progenitor cells (FLSPCs) from primary cultured fetal liver cells via density-gradient centrifugation centered on a three-step method [10].

In the present study, we aimed to determine whether HCSCs can be obtained by exploiting their physical properties. For this purpose, we applied our established three-step method with a slight adjustment for isolating HCSCs from primary HTCs. The HTCs were first isolated using Percoll discontinuous gradient centrifugation (PDGC), then purified based on differential trypsinization and differential adherence (DTDA), and finally layered by Percoll continuous gradient centrifugation (PCGC). HTCs were thus separated into four subpopulations. The third subpopulation, which contained the fewest cells, with a density varying between 1.041 and 1.062 g/ml, showed the highest expression of stem cell markers, the greatest ability to proliferate and form colonies *in vitro*, the greatest capacity to migrate *in vitro*, the strongest capacity to be resistant to chemotherapy, and the richest ability to generate tumors in NOD/SCID mice. All of these results indicated that the population from the third layer of the PCGC gradients possessed CSC characteristics. This new strategy for isolating HCSCs will make great contributions in two main areas: it will provide new insights related to the isolation of other kinds of CSCs; and it will allow us to investigate the contribution of HCSCs to HCC.

## Materials and Methods

### 1 Procedure for separating HTCs

#### 1.1 Construction of an HCC model by diethylnitrosamine (DEN) induction

Twelve male Fisher 344 rats (National Rodent Laboratory Animal Resource, Shanghai, China) in the trial group were treated with 0.05% DEN (Sigma Co, NY, USA) in their drinking water for 6 weeks and were then switched to normal drinking water [11]. Another twelve male Fisher 344 rats in the control group were given a normal diet. At 10, 14 and 18 weeks after DEN induction, four rats from each group were sacrificed. Samples of liver tissue from each group were routinely processed and stained with hematoxylin and eosin (H&E) for histological examination. All animal experiments were performed strictly according to animal study protocols [12] and approved by the Research Animal Care and Use Committee at the Fourth Military Medical University.

#### 1.2 Identification of HCC by immunohistochemistry

To further identify the type of liver tumors that occurred in the rats, the expression levels of AFP and CK19 were examined immunohistochemically. Paraffin-embedded sections were dewaxed using xylene and an ethanol concentration series, blocked with 20% goat serum, and stained with a primary rabbit-anti-rat AFP or CK-19 antibody (dilution 1∶200; Santa Cruz, CA). Goat anti-rabbit IgG secondary antibodies were used for staining AFP or CK-19. These sections were also counterstained with 2-(4-diamidinophenyl)-6-indolecarbamidine dihydrochloride (DAPI) (dilution 1∶100; Sigma) for detecting nuclei. Negative controls were stained without primary antibodies. Stained sections were examined under a fluorescent microscope (FV1000MPE, Olympus Corporation, Tokyo, Japan). The positive rates for AFP and CK-19 were determined in all sections.

#### 1.3 Isolation of HTCs by PDGC

Individual HCC nodules were removed from each rat in the trial group. Raw HTCs were isolated according to Hohne et al. [13] with minor modifications. Briefly, the HCC nodules were minced in William's E medium (Sigma Co, Louis, MO, USA) with 0.1% collagenase type IV and 0.005% trypsin (Sigma Co, Louis, MO, USA) and then incubated for 20 min at 37°C in a shaking water bath. After incubation, the released cell supernatants were passed through a 100 µm nylon mesh and centrifuged at 1,000× g for 8 min. The pellets were washed twice with phosphate-buffered saline (PBS) (Invitrogen Co, USA) to obtain raw HTCs. These raw HTCs were prepared as single cell suspensions and separated into different fractions using PDGC (30%, 50% and 70% Percoll). The cells at the interface of 30% and 50% Percoll were collected and cultured in 6-well plates containing William's E medium supplemented with 10% vol/vol fetal bovine serum (Invitrogen Co, USA), 5 µg/ml insulin (Sigma Co, Louis, MO, USA), 5 µM hydrocortisone, 100 U/ml penicillin and 100 µg/ml streptomycin at 37°C in a humidified atmosphere containing 5% CO_2_. When the adherent cells extended as a monolayer colony 20 days later, we collected the monoclonal cells by local digestion with cloning cylinders and transferred them to a new culture dish to continue the culturing process.

#### 1.4 Purification of HTCs by DTDA

To obtain pure HTCs, cells in culture were treated as follows. Based on the results of a former study by our group [10], cells of different sizes can be trypsinized within distinct time periods. It has been found when the cells are digested using 0.25% trypsin for 10 min, very large cells are trypsinized and excluded, whereas other cells remain. This method is referred to as “differential trypsinization”. Interestingly, when the cells were completely trypsinized for subculturing over a period of 120 min, large cells did not adhere to the plates and were easily removed. This phenomenon is referred to as “differential adherence”. HTCs were purified via these two stepsHTCs.

#### 1.5 Separation of HTCs by PCGC

**Figure 1:**
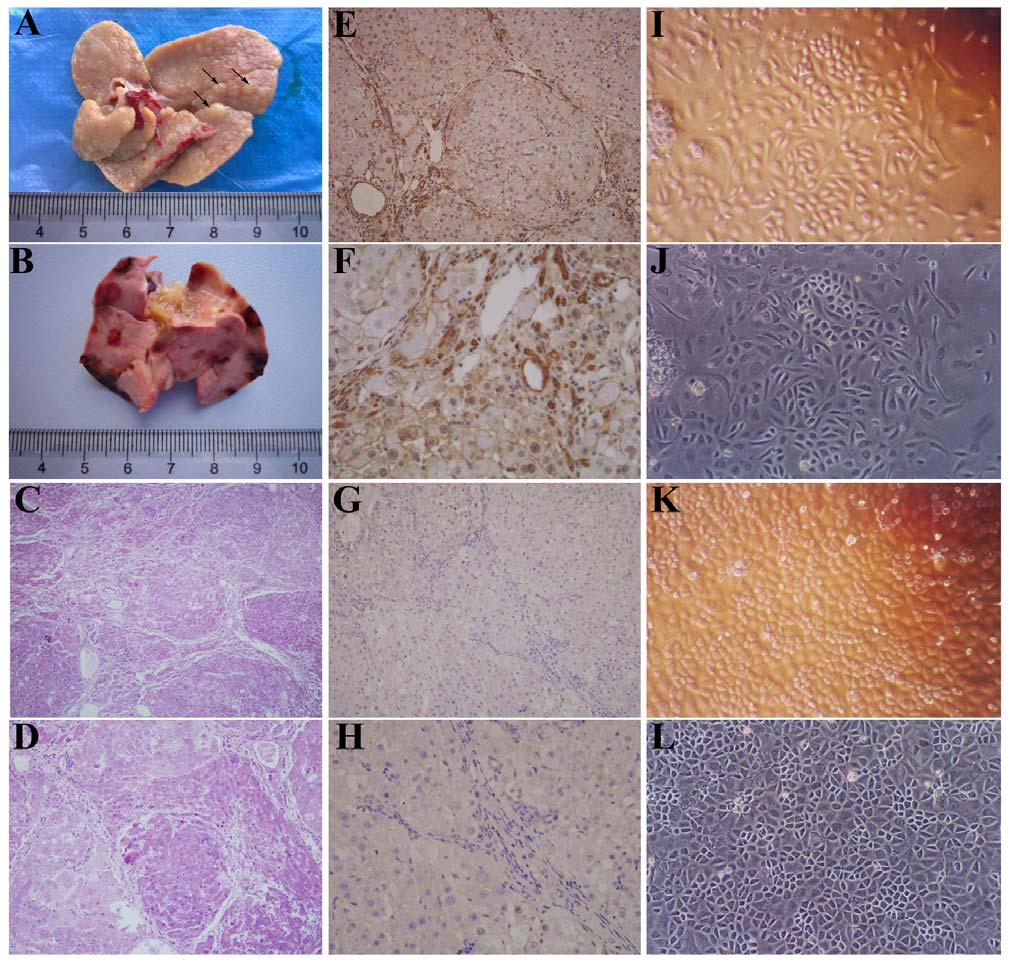
Formation of HCC and isolation of HTCs. (A) Small tumors (black arrows) were caused by 10 weeks of DEN induction. (B) The liver had been almost completely replaced by large tumors 18 weeks after DEN induction. (C) Low magnification view of an H&E-stained section from tumor tissue. (D) High magnification view of an H&E-stained section. (E) Low magnification view of an AFP-stained section. (F) High magnification view of an AFP-stained section. (G) Low magnification view of a CK-19-stained section. (H) High magnification view of a CK-19-stained section. (I, J) Images of primary cultured HTCs. (K, L) Images of HTCs purified by DTDA. Original magnifications: 100× (C, I–L), 200× (D, E, G), 400× (F, H).

The protocol was performed according to the methods of our previous study [10]. Briefly, to create continuous gradients, a 40% Percoll solution was centrifuged at 20,000×g for 90 min using an angle head rotor. The tube was then allowed to stand for 30 min, and the cell suspension was gently layered on top of the preformed gradients. The tube containing the cells was centrifuged at 500×g for 15 min using a swinging bucket rotor. From the top to the bottom of the tube, as the cell densities continuously increased, we collected four cell fractions and designated them fractions I–IV (FI–IV) (Figure 1A).

### 2 Morphological comparison among the different fractions

#### 2.1 Transmission electron microscopy (TEM)

The freshly isolated cells from each fraction were fixed with 2.5% glutaraldehyde at 4°C for 4 h, then rinsed with cold PBS and further fixed in 1% osmium tetroxide at room temperature for 2 h. The samples were first dehydrated using a gradient of ethanol and acetone, then embedded in Epon812 resin and acetone (v/v, 1∶1) for 30 min, followed by 100% Epon812 resin for 1 h. The Epon812 resin was solidified at 37°C for 24 h and at 60°C for 48 h. Ultrathin sections were prepared using an LKB ultramicrotome (Ultrotome NOVA; LKB, Broma, Sweden) and stained with uranyl acetate and lead citrate for examination by TEM (JEM-2000Ex, JEOL Ltd., Japan).

#### 2.2 Immunofluorescence (IF) staining with Phalloidin

To further analyze the morphological differences among the freshly isolated cells from each fraction, phalloidin staining was used. The cells were first treated with a primary phalloidin antibody (dilution 1∶100; Santa Cruz, CA) and then with a fluorescent FITC-conjugated goat anti-rabbit secondary antibody (dilution 1∶100; Santa Cruz, CA) for 2 h. The gross morphology of the cells was observed under a fluorescence microscope, and the numbers of cilia and pseudopodia were calculated.

### 3 Comparison of markers among different fractions

#### 3.1 Flow cytometry (FCM)

The freshly isolated cells from each fraction were prepared at a concentration of 10^6^cells/ml using William's E medium (containing 20% FBS) and incubated for 15–30 min at room temperature to block non-specific sites. These cells were then washed twice with PBS and re-suspended in 990 µl PBS. Subsequently, 10 µl of antibodies, including CD133 (PE-conjugated, Biolegend, USA) and EpCAM (FITC-conjugated, Biolegend, USA), were added to each cell suspension. After 30 min of incubation at 4°C in the dark, the cells were washed twice with PBS, fixed in 0.1% formaldehyde and analyzed using the FACSCaliburTM system (BD Immunocytometry Systems, San Jose, CA).

#### 3.2 IF analysis

After the freshly isolated cells from each fraction adhered to a culture slide, they were washed twice with PBS, fixed with 4% paraformaldehyde for 20 min, and immersed in PBS for 10 min, followed by exposure to 0.01% Triton X-100 at room temperature for 10 min. The cells were treated with 6% goat serum (Santa Cruz, CA) at room temperature for 30 min to block non-specific immune reactions and then with primary CD133 (dilution 1∶200; Santa Cruz, CA), AFP (dilution 1∶200; Santa Cruz, CA) and CK-19 (dilution 1∶200; Santa Cruz, CA) antibodies at 4°C overnight. After the samples were washed twice with PBS, they were incubated with fluorescent PE/FITC-conjugated goat anti-rabbit secondary antibodies (dilution 1∶100; Santa Cruz, CA) for 2 h. Subsequently, the samples were treated with DAPI (dilution 1∶100; Sigma) for 15 min. The fluorescence of the samples was observed using a fluorescence microscope (FV1000MPE, Olympus Co, Tokyo, Japan).

#### 3.3 Quantitative real-time polymerase chain reaction (qRT-PCR)

Total RNA was extracted from the freshly isolated cells of each fraction using the TRIzol reagent (Molecular Research Center, Cincinnati, OH) and reverse transcribed into cDNA with SuperScript II Reverse Transcriptase according to the manufacturer's instructions (Invitrogen, Carlsbad, CA). An equal amount of cDNA from each sample was amplified with specific primers (Table 1). PCR was carried out using SYBR Green Master Mix (Applied Biosystems, Foster City, CA) over 40 cycles. The cycling parameters for the PCR were 10 minutes at 95°C, 15 seconds at 95°C, and 30 seconds at 60°C, with a final dissociation step of 15 seconds at 95°C, 20 seconds at 60°C, and 15 seconds at 95°C using the Bio-Rad MyIQ5 real-time PCR system (Bio-Rad, California, USA). Each sample was assayed in triplicate. mRNA levels were normalized using GAPDH as an internal reference.

**Table 1:** The primers used in the QRT- PCR.

Gene Name		Sequence	*Tm* (°C)	Product length
CK-19	Forward	aaccacgaggaggaaattagtg	59.5	225
	Reverse	tatctggatctgcgtagtgtgg	60.2	
AFP	Forward	ctgtatgctcccaccattcttt	60.4	109
	Reverse	ttgatgctctctttgtctggaa	60.0	
ALB	Forward	gacaaagcagcctgcctgac	63.4	174
	Reverse	ttctgcgaactcagcattgg	62.5	
EpCAM	Forward	gtcattgtcgtggtggtgttag	60.3	109
	Reverse	cacccatctcctttatctcagc	60.1	
GAPDH	Forward	atggtggtgaagacgccagta	55.5	143
	Reverse	ggcacagtcaaggctgagaatg	55.9	

### 4 The induced differentiation by hepatocyte growth factor (HGF)

All the cells from each fraction were separately cultured in commercial serum-free medium (Sigma Co, Louis, MO, USA) supplemented with HGF at a concentration of 20 ng/ml. After all the cells in 96-well plates (1×10^3^ cells/well) were cultured for 14 d, they were collected and the exact identification was performed as follows. The differentiation was evaluated by detecting the expression of CSCs specific marker CD133 by FCM and HCC marker AFP by IF. The detailed protocols were as described above.

### 5 Functional comparison among different fractions

#### 5.1 3-(4, 5-dimethylthiazol-2-yl)-2, 5-diphenyltetrazolium bromide (MTT) assay

The proliferative abilities of the freshly isolated cells from each fraction were determined using the MTT assay. Briefly, the cells were cultured in 96-well plates at a density of 1×10^3^ cells/well. At 2, 4, 6, 8, 10, 12 and 14 days of culture, MTT (Sigma, St. Louis, MO, USA) dissolved in PBS was added to each well at a final concentration of 5 mg/ml, and the samples were incubated at 37°C for 4 h. Water-insoluble dark blue formazan crystals that formed during MTT cleavage in actively metabolizing cells were then dissolved in dimethyl sulfoxide (DMSO) (Gibco/Invitrogen, NY, USA). The optical density was measured at a wavelength of 490 nm with a Bio-Rad 680 microplate reader (Bio-Rad, California, USA).

#### 5.2 Colony formation test

The freshly isolated cells from each fraction were inoculated separately into each well (10 cells per well) of 96-well plates (Corning Life Sciences, Acton, MA) supplemented with 100–200 µl of William's E medium plus leukemia inhibitory factor (LIF) at a concentration of 10 µg/ml. Cell viability was assessed via staining with trypan blue (Sigma-Aldrich) at several time points. After 4 weeks of culturing, each well was examined using light microscopy, and the total number of cell colonies was counted. Images of the colonies were recorded using a microscope (Nikon Eclipse TS100, Nikon, Kingston-upon-Thames, UK) and a digital camera (C4742-95, Hamamatsu photonics, Welwyn Garden City, Hertfordshire, UK) under phase contrast conditions.

#### 5.3 Transwell migration assay

A total of 1×10^5^ cells in 0.5 ml of serum-free Williams' E medium from each fraction were seeded on an 8-µm pore-size polycarbonate membrane Boyden chamber inserted in a transwell apparatus (Costar, Cambridge, MA) coated with Matrigel. A total of 600 µl of Williams' E medium containing 20% FBS was added to the lower chamber. After incubation for 24 h at 37°C in a 5% CO_2_ incubator, the cells on the top surface of the insert were removed by wiping with a cotton swab. The cells that had migrated to the bottom surface of the insert were fixed in 100% methanol for 2 min, stained in 0.5% crystal violet for 2 min, rinsed in PBS and subjected to inspection via microscopy. Values for invasion and migration were obtained by counting under an inverted light microscope (Olympus Corporation, Tokyo, Japan) in 5 randomly selected fields.

#### 5.4 *In vitro* scratch assay

Each well of 24-well tissue culture plates was seeded with cells to a final density of 100,000 cells per well, and these cells were maintained at 37°C under 5% CO_2_ for 24 h to allow them to adhere and to form confluent monolayers. These confluent monolayers were then scored with a sterile pipette tip to leave a scratch of approximately 0.4–0.5 mm in width. The culture medium was then immediately removed (along with any dislodged cells). The removed medium was replaced with fresh William's E medium. All scratch assays were performed in triplicate. Scratch closure was monitored by collecting digital images at the beginning of the experiment and at regular intervals during the process of cell migration to close the scratch, and the images were compared to quantify the migration rate of the cells. Digital images were captured using an inverted microscope (Nikon Eclipse TS100, Nikon, Kingston-upon-Thames, UK) and a digital camera (C4742-95, Hamamatsu photonics, Welwyn Garden City, Hertfordshire, UK) under phase.

#### 5.5 Chemotherapeutic experiment

To confirm *in vitro* chemo-resistance, 1.5×10^5^ cells from each fraction were plated in 24-well plates and treated with paclitaxel (10 ng/ml) for 24 h. The optimal dose of paclitaxel used in this study was adopted according to our preliminary experiments. An Annexin V-FITC/PI apoptosis detection kit (Annexin V-FITC/PI Staining Kit; Immunotech Co.,Marseille, France) was used for the detection of cell apoptosis. Briefly, the cells treated with paclitaxel were collected, washed in cold PBS, incubated for 15 min with fluorescein-conjugated annexin V and PI according to the manufacturer's protocols, and analyzed by FCM.

#### 5.6 Tumor formation in NOD/SCID mice

Thirty-six male NOD/SCID mice (6–8 weeks old) obtained from the laboratory animal center of Beijing Xiehe Medical University (Beijing, China) were maintained in pathogen-limited conditions at the animal resources center of the Fourth Military Medical University (Xi'an, China). These mice were randomly divided into six groups: F0, FI, FII, FIII FIV, and the control HeG2 cell line. Thus, each group was supplied with 6 mice. The cells were injected into NOD/SCID mice via subcutaneous injection in different amounts (1×10^5^ and 1×10^4^). Tumor growth was monitored every 2 days after the second week of inoculation. All mice were sacrificed 60 days after injection. All of the resultant tumor tissues were collected, fixed in 4% formaldehyde, and embedded in paraffin for H&E staining to assess tumor histology. The tumors were classified according to their size. The existence of no macroscopic tumor was defined as negative (−); a tumor diameter of <0.2 cm as positive (+); a diameter of 0.2–0.5 cm as moderately positive (++); and a diameter of >0.5 cm as strongly positive (+++).

### 6 Statistical analysis

SPSS 14.0 software was used for all statistical evaluations. All data are expressed as the means ± standard error of separate experiments (n≥3, where n represents the number of independent experiments). The data were analyzed for statistical significance using one-way ANOVA. *P*<0.05 was considered statistically significant.

## Results

### 1 HCC formation and HTCs isolation

When the rats were sacrificed 10 weeks after DEN induction, only small tumors could be found (Figure 1A). At 18 weeks after DEN induction, most of the liver tissue of the rats had been replaced by tumor tissues with rough surfaces (Figure 1B). Based on the assessments of three independent pathologists, H&E staining verified that the tumors were all hepatic carcinoma derived (Figure 1C, D). To further identify the type of the tumors, we selected one marker for HCC (AFP) and another marker for cholangiocarcinoma (CK-19) to stain tissue sections from the same tumors. Most of the tumor cells within tumor nodules stained positively for AFP (68.7±6.21%) (Figure 1E, F). In contrast, almost no cells that stained positively for CK-19 were found over a large area (1.12±0.13%) (Figure 1G, H). In summary, the tumors stained positively for AFP and negatively for CK-19. This finding indicates that these tumors were almost hepatic origin, which is consistent with the results of H&E staining. The HTCs isolated from primary HCC tissues grew slowly at first, with only a few colonies being formed, and displayed heterogeneous morphology (Figure 1I, J). Through DTDA purification, HTCs proliferated rapidly and were much more homogenous (Figure 1K, L). Thus, these HTCs could be used for subsequent experiments.

### 2 Fractionation of HTCs by PCGC

**Figure 2:**
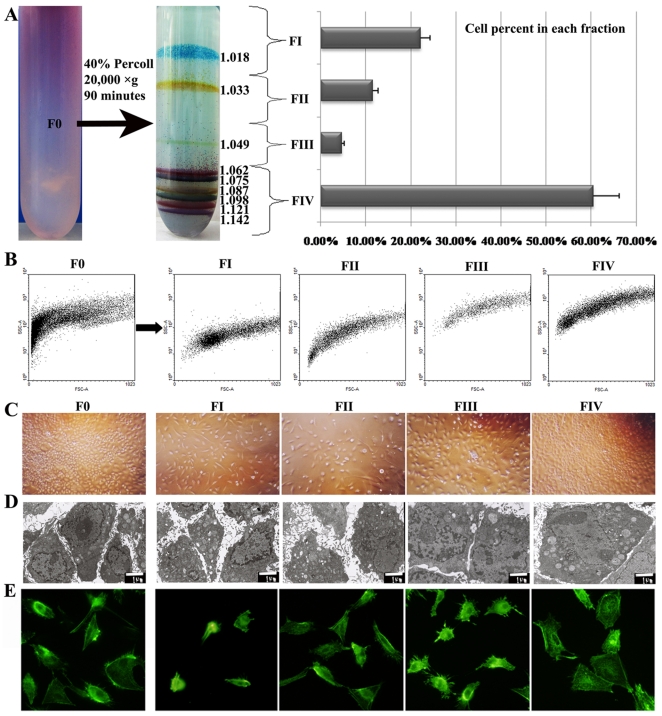
Separation of HTCs and morphological comparison. (A) Schematic illustration of the continuous Percoll gradient used for fractionation and the percentages of cells segregated in each fraction. (B) Dot-plot histograms resulting from FACS analysis showing the densities of cells in each fraction. (C) Light microscopy of cells from each fraction under phase. (D) TEM images and diameters of cells from each fraction. (E) Images of cell stained by phalloidin reflect the different morphologies of cell surfaces. Original magnifications: 100× (C), 6000× (D), 400× (E).

In a former study, we found that centrifugation of 40% Percoll for 90 min resulted in the formation of an optimal continuous gradient [10]; therefore, in the present study, we applied this methodology to fractionate HTCs. Under these conditions, according to the distribution of cell pellets, we divided the HTCs population (F0) into four main fractions (fraction I–IV, FI–IV). Based on the monitoring of Density Marker Beads, four cell fractions were gently removed individually from the tube, from the top to the bottom (Figure 2A). Cell counting revealed that 22.18±2.13%, 11.62±1.06%, 4.73±0.38% and 61.47±6.24% of the cells were segregated in FI–FIV, respectively, meaning that FIV contained the most cells, and the fewest cells (less than 5% cells) were found in FIII (Figure 2A). Based on dot-plot histograms obtained from FACS, before separation, the cells in F0 were heterogeneous in size, whereas after separation, the size of the cells in each fraction became somewhat more homogenous. As expected, FCM analysis of each subpopulation revealed an increase in cell granularity [FSC (forward scatter)/SSC (side scatter)] with increasing Percoll density (Figure 2B). Thus, from FI to FIV, the cells continually increased in size.

### 3 Morphological comparison of cells from FI-FIV

The cells from each fraction were cultured separately in William's E medium. The cells from FIII were the first to adhere to plates, which took place in less than 2 h, followed by the cells from FI and FII in 2.5 h, and the cells from FIV and F0, which required more than 3 h. Under *in vitro* culture conditions, the heterogeneously sized cells in F0 could be easily observed. After separation, it was found that the cells from FI and FII were the smallest, followed by FIII, and the cells from FIV were the largest (Figure 2C).

The TEM images displayed similar phenomena as observed in the results obtained via light microscopy. The F0 cells exhibited a great variety of diameters, ranging from 6 µm to 30 µm (Figure 2D). The diameters of the cells from FI and FII were the smallest, followed by FIII, and the diameters of cells from FIV were the largest (Figure 2D). Meanwhile, the cells from FIII presented the highest nuclear-cytoplasmic ratio and the fewest organelles; in contrast, the cells from FIV showed the lowest nuclear-cytoplasmic ratio and the most organelles (Figure 2D). This indicated that the cells from FIII were the most immature.

In addition, we stained cells from each fraction using phalloidin. Under fluorescence microscopy, the cells of FIII were slightly more brightly stained by phalloidin and displayed more cilia or pseudopodia on their surfaces than did the cells of any other fraction (Figure 2E). However, the statistical analysis indicated the difference was not significant (*P*>0.05).

### 4 Expression of stem cell markers

**Figure 3:**
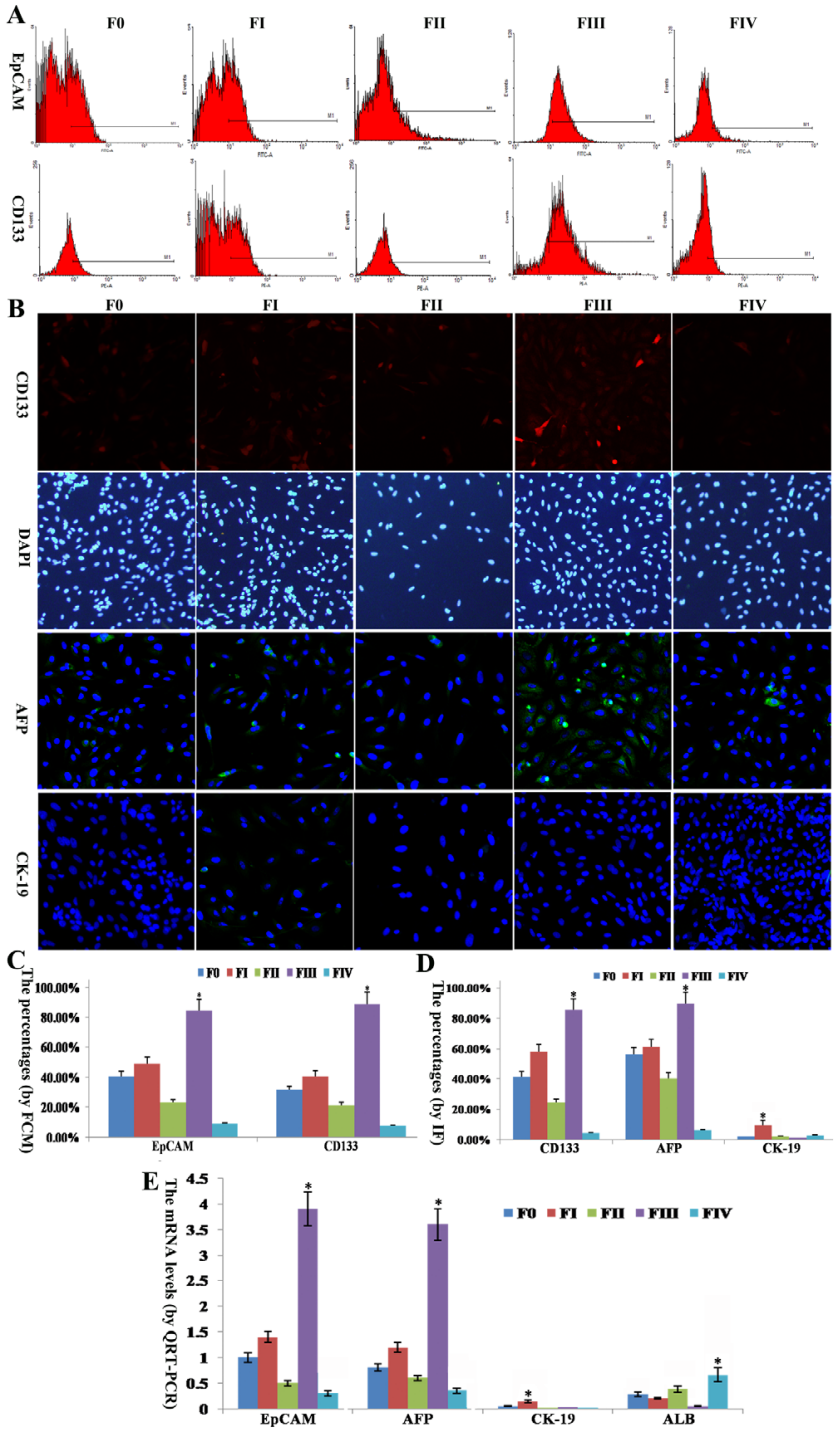
Expression of specific markers in each cell fraction. (A) Expression of stem cell markers (EpCAM and CD133) in each fraction determined by FCM. (B) Based on IF, CD133-positive cells (red, nuclei in blue) and AFP and CK-19-positive cells (green, nuclei in blue) were found to be included in different proportions. (C) Column chart reflecting the data obtained via FCM. (D) Column chart reflecting the data generated by IF. (E) qRT-PCR results of specific markers in freshly isolated cells from each fraction. F0 represents unfractionated HTCs; FI-IV indicates fractions I–IV after separation. * refers to *P*<0.01 compared with any other fraction. Original magnification: 100× (B).

To determine the stem properties of cells in each fraction, two relatively widely accepted stem cell markers were detected by FCM immediately after separation. Both EpCAM and CD133 were most highly expressed in the freshly isolated cells from FIII compared with the other fractions (*P*<0.01) (Figure 3A, C). The percentage of EpCAM-positive cells in FIII was as high as 81.5±7.96%, and the percentage of CD133-positive cells was even higher, at 84.7±8.53%. In contrast, the expression of both stem cell markers in freshly isolated cells from FIV was lowest: 7.1%±0.64% for EpCAM and 5.7%±0.63% for CD133 (Figure 3A, C).

To further identify the stem properties of cells from each fraction, we detected the expression of CD133 using IF. The percentage of CD133-positive cells was the highest in FIII, at 83.2%±8.01%, and the lowest in FIV, at 4.6%±0.42%, (Figure 3B, D). We also selected one hepatic tumor marker (AFP) and one biliary tumor marker (CK-19) to identify cells from each fraction. After segregation, the percentage of AFP-positive cells in FIII was the highest (89.6%±8.27%), being much higher in any other fraction (*P*<0.01) (Figure 3B, D). However, only a few cells in FI were determined to be CK-19 positive (6.1%±0.54%), and almost no cells in F0, FII, FIII and FIV expressed CK-19 (Figure 3B, D).

The phenomenon described above was quantitatively verified using qRT-PCR. The relative levels of EpCAM and AFP mRNA were highest in cells from FIII (*P*<0.01), being nearly four times as high as observed in cells from F0. In contrast, the EpCAM and AFP mRNA levels were lowest in cells from FIV (*P*<0.01), being half of the levels in cells from F0 (Figure 3E). In accord with the results of IF, CK-19 mRNA could only be weakly detected in cells from FI (relative fold was 0.103±0.012); however, CK-19 mRNA could barely be detected cells of F0, FII, FIII and FIV(Figure 3E). Additionally, the mature hepatic marker ALB was moderately expressed in cells from FIV, very weakly expressed in cells from F0, FI and FII, and expressed almost not at all in cells from FIII. It can be speculated that the cells from FIII were much more immature than cells from any other fraction (*P*<0.01).

### 5 The induced differentiation of cells from each fraction

**Figure 4:**
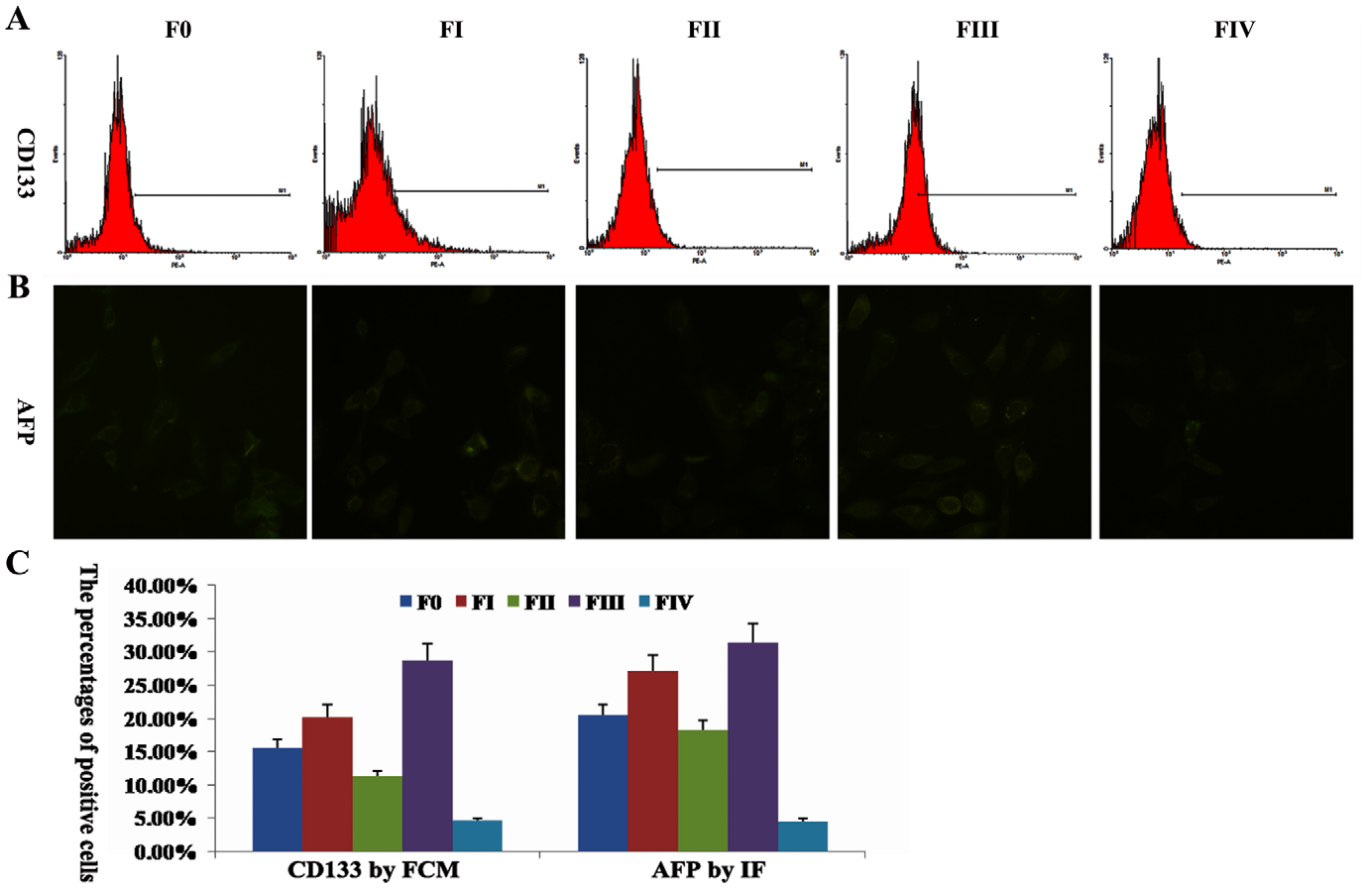
The differentiation of cells from each fraction induced by HGF. (A) Expression of stem cell marker CD133 in daughter cells from each fraction determined by FCM. (B) Based on IF, AFP-positive cells (green) were found in daughter cells from each fraction. (C) Column chart reflects the data of positive cells. F0 represents unfractionated HTCs; FI-IV indicates fractions I–IV after separation.

Before induction, the cells from FIII were homogeneous in morphology, when these cells were induced by HGF, they proliferated very slowly, and many heterogeneous cells were observed. Before induction, the cells from FIII expressed CSCs marker CD133 (84.7±8.53%) higher than cells from other fractions, 14 days after induction, the daughter cells from FIII expressed CD133 as low as 28.7±3.02%, which was almost the same as daughter cells from F0 and FI did (Figure 4A, C). In addition, induced by HGF, the cells from FIII generated weak AFP-positive cells. Although the percentage of AFP-positive cells was still higher in daughter cells of FIII than in other fractions, the daughter cells from FIII expressed AFP as low as 31.8±3.15% (Figure 4B, C). That is to say, after induction the level of AFP expression in FIII was decreased by 1.9 times. The above results indicate that cells from FIII could be induced to lose CSCs-like properties and differentiate into mature HTCs.

### 6 Proliferative abilities of cells from each fraction

**Figure 5:**
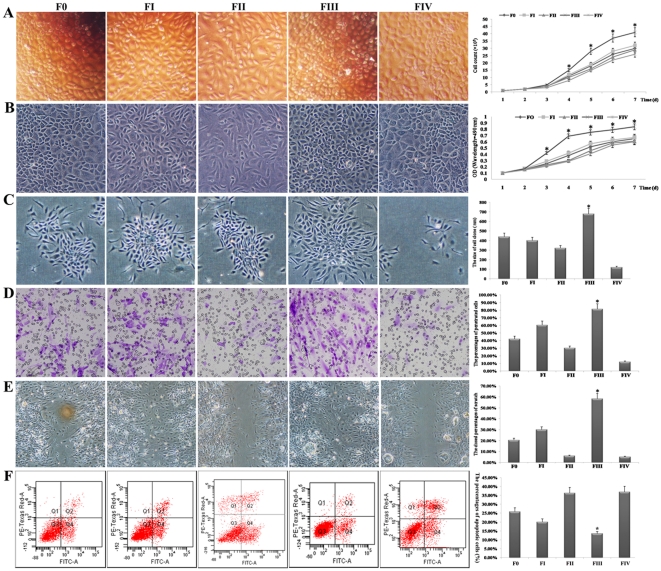
Proliferative and invasive capacities of cells from each fraction. (A) Left panel: light images of cells from each fraction after 7 d culture; right panel: column chart of cell counts during the cell culture period. (B) Left panel: gray images of cells from each fraction after 7 d culture; right panel: results of MTT assays during the cell culture period presented in a column chart. (C) Left panel: gray images of cell colonies generated by cells from each fraction; right panel: average diameters of cell colonies summarized in a column chart. (D) Left panel: light images of penetrating cells from different fractions in transwell assays; right panel: average percentages of cells that crossed the membrane are shown using a column chart. (E) Left panel: grayscale images of scratch assays showing the different abilities among cells from different fractions to close scratches via migration; right panel: average percentages of closed scratches are provided in a column chart. (F) Left panel: the apoptotic analysis for cells from each fraction by FCM; right panel: the percentages of apoptotic cells are shown by a column chart. F0 represents unfractionated HTCs; FI-IV indicates fractions I–IV after separation. * refers to *P*<0.01 compared with any other fraction. Original magnification: 200× (A–D), 100× (E).

One primary characteristic of CSCs is rapid proliferation. During 7 days of culture, cells from FIII proliferated the most rapidly, followed by cells from FI and F0, while cells from FII and FIV proliferated slowest. Because there were the same number of initial cells in each fraction, it was possible to observe in a column chart of cell counts that beginning on the 4^th^ day, the number of cells in FIII was much higher than in any other fraction (*P*<0.01) (column charts shown in Figure 5A). Accordingly, the cell density in FIII was the highest, and these cells were relatively more homogeneous than cells from other fractions (light images in Figure 5A). MTT assays showed that beginning on the 3^rd^ day, the OD value generated by cells from FIII was higher than those produced by cells from the other fractions, and the gap in OD values between cells from FIII and cells from all other fractions became increasingly larger (column charts in Figure 5B). This was in strict agreement with what was observed in the grayscale images of cells from each fraction on the 7^th^ day of culture (Figure 5B).

Another identifying characteristic of CSCs is their ability to form cell colonies. Under the same culture conditions, the cells from FIII formed colonies first, followed by cells from FI and F0, and the cells from FII and FIV generated colonies the most slowly. After 7 days of culture, not only the number of colonies but also the average diameters of the colonies produced from each fraction differed greatly (Figure 5C). For example, the average diameters of the cell colonies were as follows: 440 µm for F0, 400 µm for FI, 320 µm for FII, 680 µm for FIII, and 120 µm for FIV (Figure 5C). Because the cells from FIII showed the greatest ability to form cell colonies, it could be speculated these cells were more likely to be HCSCs.

### 7 Migration abilities of cells

Based on the results of phalloidin staining, it was speculated the cells of FIII would be a little more invasive than cells from other fractions. The results of transwell assays indicated that under the same incubation conditions, cells from FIII displayed significantly higher transmembrane activity than did cells from any other fraction (*P*<0.01). The percentage of cells that penetrated from FIII was as high as 81.50%±7.35%. In contrast, the percentage of cells that transited the membrane among FIV cells was as low as 12.13%±1.21% (Figure 5D). To shed further light on this phenomenon, we performed scratch assays. Two days after scratches were produced, although they were not closed by the cells from any fraction, the closed percentage of the scratches varied widely among the different fractions. Interestingly, the percentage of closed scratches generated by cells from FIII, which was 58.4%±5.02% (Figure 5E), was much higher than that produced by other fractions (*P*<0.01). These data demonstrate that cells from FIII exhibited a very high capacity for invasion and migration.

### 8 Resistance to chemotherapy

Because chemo-resistance is associated with CSCs-like phenotype [14], the cells from each fraction were evaluated by paclitaxel treatment. We assessed the apoptotic rate of cells in each group and summarized the results in Table 2. The results suggested that there were significantly more normal cells and fewer apoptotic cell from FIII than from any other fraction (*P*<0.01). The percentage of apoptotic cells from FIII, including early and late apoptosis, was as low as 13.50%±1.21%. In contrast, the percentage of apoptotic cells from FIV was as high as 36.91%±3.18% (Figure 5F).

**Table 2:** The apoptosis of cells from each fraction after paclitaxel treatment (%).

Fractions	Normal	Early apoptosis	Late Apoptosis	Necrosis
F0	73.56±2.16*	21.54±1.02*	4.18±0.32	0.62±0.03
FI	79.45±2.29	15.55±1.13	4.43±0.34	0.47±0.02†
FII	56.54±2.21*	29.06±1.06*	7.28±0.52*	7.12±0.64*
FIII	86.12±2.14	11.09±0.86	2.42±0.58	0.38±0.02
FIV	58.00±2.05*	22.10±1.16*	14.81±1.18*	5.19±0.35*

Note:

* *P*<0.01 compared to FIII;

† *P*>0.05 compared to FIII.

### 9 Tumorigenic potential of cells from each fraction

**Figure 6:**
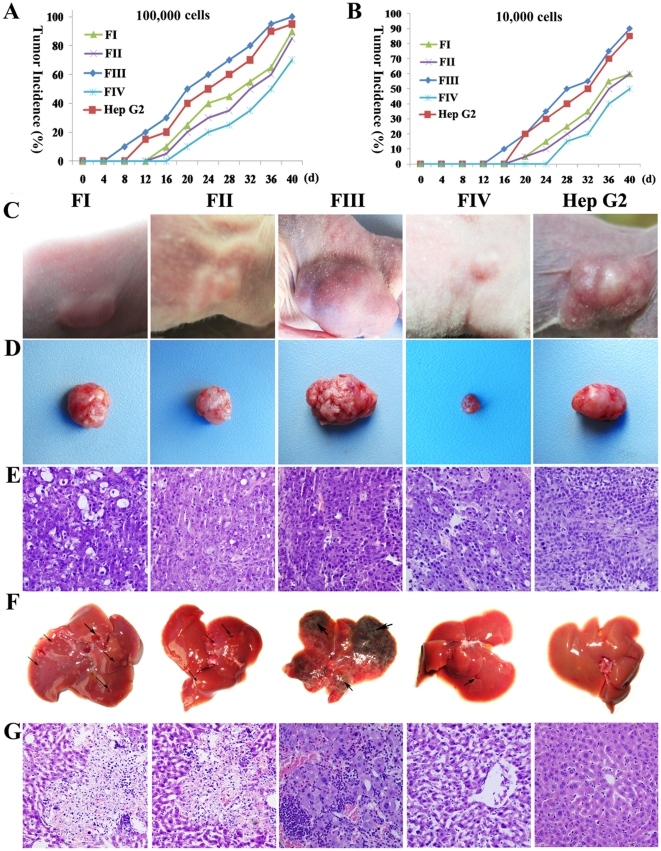
Tumorigenic and invasive potential of cells from each fraction. (A) Column chart showing the tumor incidence in each group following the injection of 1×10^5^ cells. (B) Column chart showing the tumor incidence in each group following the injection of 1×10^4^ cells. (C) Subcutaneous tumors in mice following the implantation of 1×10^4^ cells from each group. (D) Representative tumors were selected accordingly. (E) Histological analysis of subcutaneous tumors by H&E staining, showing HCC-specific features (irregularly packed tumor cells, hyperchromatic nuclei, high density of microvessels and pleomorphism). (F) The livers of the mice were damaged to different extents by injecting 1×10^4^ cells from each group (black arrows). (G) H&E staining of liver lesions. Original magnification: 200× (E, G).

The growth of a subset of tumor cells (typically less than 5% of total tumor cells) in immunodeficient mice has become the “gold standard” for identifying CSCs [15]. To test the tumorigenic ability of cells from each fraction, various numbers of cells were injected into mice. After the number of tumors in each mouse was counted, the size of each tumor was measured, and liver metastasis in each mouse was examined; the data obtained from these analyses are summarized in Table 3. Generally speaking, when 1×10^5^ cells were used, cells from F0, FI, FIII and HepG2 were able to form tumors in every mouse; however, the same number of injected cells from FII and FIV were able to generate tumors in only half of the mice. Additionally, the average size of tumors formed by cells from FIII was much larger than associated with cells from any other group (*P*<0.01) (Figure 6A). Furthermore, liver metastasis was most easily detected in mice injected with cells from FIII (Table 3). When 1×10^4^ cells were used, xenograft tumors were found within nearly every mouse injected with cells from FIII, whereas the proportion of mice with tumors was 4/6 for FI and HepG2, 3/6 for F0, and only 1/6 for FII and FIV (Table 3, Figure 6B). As few as 1×10^4^ cells from FIII could initiate tumors not only in subcutaneous tissues (Figure 6C, D) but also in liver tissues of NOD/SCID mice (Figure 6F). Pathological analysis indicated that the tumors from the subcutaneous regions (Figure 6E) and tumors from livers (Figure 6G) were all hepatic carcinoma derived. However, the same number of cells (1×10^4^) from the HepG2 cell line did not cause damage to the liver, including failing to generate tumors in liver tissue (Figure 6F, G) in NOD/SCID mice. In summary, when the same number of cells was used, cells from FIII caused larger tumors and more frequent liver metastasis than did cells from the other fractions (*P*<0.01).

**Table 3:** The tumorigenic potential of cells from each fraction.

Cell subpopulations	Tumor incidence	Tumor diameter	Metastasis incidence
F0 (1×10^5^)	6/6	**++**	2/6
FI (1×10^5^)	6/6	**++**	2/6
FII (1×10^5^)	3/6	**+**	0/6
FIII (1×10^5^)*	6/6	**+++**	6/6
FIV (1×10^5^)	3/6	**+**	0/6
HepG2 (1×10^5^)	6/6	**++**	0/6
F0 (1×10^4^)	3/6	**+∼++**	2/6
FI (1×10^4^)	4/6	**+∼++**	2/6
FII (1×10^4^)	1/6	**−∼+**	1/6
FIII (1×10^4^)*	6/6	**+++**	5/6
FIV (1×10^4^)	1/6	**−∼+**	1/6
HepG2 (1×10^5^)	4/6	**++**	0/6

“Tumor incidence” indicates the average incidence of tumors in each mice (6 mice in each group, 6/6 means 6 tumors in 6 injected mice). “Tumor diameter” refers to the average diameter of tumors in each group (−, no macroscopic tumor; +, <0.2 cm; ++, 0.2–0.5 cm; +++, >0.5 cm). “Metastasis incidence” means the average incidence of the liver neoplasia in each group (6/6 means 6 liver neoplasias found in 6 mice). All results were independently viewed by three different researchers.

* FIII *vs.* F0, FI, FII, FIV (the same number), *P*<0.01.

## Discussion

Recent evidence indicates that tumors contain a small population known as cancer stem cells (CSCs) that are responsible for tumor initiation, maintenance and spreading [16]. The American Association of Cancer Research Workshop working group presented a consensus definition of the CSCs as “cells within a tumor that possess the capacity for self-renewal and that can cause the heterogeneous lineages of cancer cells that constitute the tumor” [17]. Although the field of CSC biology is relatively young, continued elucidation of the features of these cells holds promise for the development of novel patient therapies [18]. Although evidence has been provided to support the existence of CSCs in various solid tumors, the isolation of HCSCs has not been clearly demonstrated.

Currently, various techniques based on the immunochemical and physical characteristics of stem/progenitor cells are used for enrichment of these cells types. Among these methods, FACS or magnetic bead separation, which are both based on cell surface markers, are most commonly used for either positive or negative selection, respectively [19]. Among these markers, the same specific marker can be used to isolate CSCs from different types of cancers, whereas different markers can also be used to isolate CSCs from the same types of cancers [20]. For example, EpCAM is over-expressed in most solid cancers and has recently been identified as a CSC marker [21]. CD133 is among the most useful markers for the identification of CSCs, including those involved in colorectal cancer [22], lung cancer [23], glioblastoma [24], [25], and thyroid cancer [26]. It appears that CD133 is a widely used marker for CSCs; however, it is not highly specific for each kind of cancer. For example, CD44 has been demonstrated to be more specific than CD133 for isolating gastric cancer-initiating cells from a panel of human gastric cancer cell lines [27]. However, CD44 is still not generally applicable for CSC detection in many cases [3], [28]–[32]. In addition, it should be noted that there exist some discrepancies regarding CSC markers among different groups [6], [29], [33]–[40]. As recently reported [41], CD133 may not be a true marker for colon CSCs, although two earlier studies independently described CD133 as a good CSC marker for colon cancer [6], [35]. Thus, a single marker may not be sufficient to isolate CSCs, and as a result, several markers are employed together to isolate certain types of CSCs, such as CD44+CD133+CD24+ cancer stem-like cells involved in murine melanoma [42], [43], CD133+CD34+ melanoma CSCs [44], and CD133+/nestin+ lung cancer CSCs [45]. Furthermore, these immunochemical methods present the disadvantages of being time consuming and expensive because of the need to use monoclonal antibodies and advanced technology. In addition, although greater purity is obtained via immunological assays for isolating cells that express a particular surface marker, the resulting cell populations are still heterogeneous.

To avoid the above limitations, the alternative approach of using SP cells has been applied for the enrichment of CSCs in some organs and tissues [46], such as skeletal muscle, breast, liver, small intestine, and uterus [47]–[51]. With respect to a possible relationship between SP fractions and CSCs, an SP fraction was also found associated with HCC [52]. Although SP cells have been isolated from HCC, the association between the SP fraction and HCSCs has not been confirmed. Furthermore, as reported by other investigators [53], we observed that Hoechst 33342 staining, which is used in FACS to isolate SP cells, was harmful to HTCs [9]. Therefore, non-SP cells may also harbor cancer stem-like cells. These results imply the limitation of using SP cells from HCC for the isolation of HCSCs. Thus, other effective approaches should be sought to isolate HCSCs.

The physical separation of CSCs remains a good alternative. Similar to the results of another study [54], we hypothesized that the unique features of CSCs may allow them to be isolated based on their specific morphology. Because HTCs exhibit different levels of granularity, we considered the possibility of exploiting properties related to density to separate these cells. Density-gradient centrifugation (DGC) is an established physical method for separation that has been used for years by investigators to facilitate the isolation of different cell types [55]. Percoll has been determined to be an ideal medium for these experiments, as it is a well-known reagent that is relatively inexpensive and easy to acquire [56]. This medium can be diluted to produce a wide range of densities without difficulty. As HCSCs had not previously been characterized based on density, we applied our established three-step method [10] for enriching HCSCs. HTCs were first isolated using PDGC, purified via DTDA, and finally separated into four fractions with PCGC. To verify the efficiency of our method, we examined the self-renewal and multiple potentials of cells from each fraction. All of the results of these analyses showed a preferential distribution of CD133 and EpCAM in a population characterized by a density between 1.041 and 1.062 g/ml (FIII), which included most CSC-like cells.

More specific isolation of HCSCs will shed new light not only on the cellular origin of HCC but also on the underlying biological mechanisms associated with this specific CSC population, which will ultimately lead to the development of more specific therapeutic agents for the treatment of this deadly cancer [43]. Three years ago, we found that reactivated CD133-positive cells were frequently present in HCC [57], corresponding to later-stage tumors. Subsequently, several studies used CD133 to isolate HCSCs [58]–[61]. To date, although several possible markers for HCSCs, including CD133 [22], [58]–[61], EpCAM [8], [21], [62], CD90/CD44 [61], DLK [63], CD24 [62], [64], CD13 [65] and OV6 [66], have been used to isolate putative CSCs from HCC in different studies, it is unknown which of these may be a “one-fits-all” marker for CSCs in HCC. Therefore, the presence of markers alone should be viewed with caution in isolating HCSCs [60], [62], [67].

In this study, we developed a novel methodology that allowed efficient enrichment of CSCs from primary cultures of HTCs. To ensure the purity of HTCs, we employed DTDA to remove non-target cell types. Using PCGC, a small subset of HTCs (less than 5% of total HTCs) was enriched in FIII, where the density ranged from 1.041 to 1.062. These cells were observed to resemble CSCs in many aspects. First, these cells possessed the capacities of self-renewal and to form cell colonies derived from single parental cells. The morphology of cells from FIII was similar to CSCs, such as exhibiting high nuclear-cytoplasmic ratio, few organelles, many cilia or pseudopodia, cellular pleomorphism, and hyperchromatic nuclei. Second, the cells from FIII expressed high levels of stem cell markers, such as AFP, EpCAM and CD133. Third, the cells from FIII could greatly differentiate into mature HTCs by HGF induction. Fourth, based on scratch assays and transwell assays, the cells from FIII showed the greatest capacity to close scratches and penetrate wells, indicating that these cells were most likely to migrate. Fifth, through chemotherapeutic experiment, the cells from FIII were most resistant to paclitaxel, which might be associated with CSCs-like phenotype. Finally, using the “gold standard” to identify CSCs [15], the cells from FIII showed the greatest ability to form tumors in both subcutaneous and liver tissues in immunodeficient mice. Taken together, our findings confirmed that FIII contained a population of cells with characteristics resembling those of CSCs. The cells from FIII were potentially cancer stem-like cells with characteristics of *in vitro* self-renewal, multilineage differentiation potential, and an *in vivo* tumorigenic capacity.

### Conclusions

In conclusion, we have shown that HTCs within the moderate-density interface between 1.041 and 1.062 g/ml contained cells with stem-like properties. Although these enriched cells might be heterogeneous, they contained a greater amount of HCSCs. The method described in the present paper provides an alternative to existing separation methods (FACS, MACS, SP) for the isolation of HCSCs. We anticipate that this study will encourage and enable investigators to move forward in HCSC research, which will aid in exploring the composition and origin of HCSCs and the molecular mechanisms underlying their genesis.
